# Analytical and toxicological evaluation of flavor chemicals in electronic cigarette refill fluids

**DOI:** 10.1038/s41598-018-25575-6

**Published:** 2018-05-29

**Authors:** Rachel Z. Behar, Wentai Luo, Kevin J. McWhirter, James F. Pankow, Prue Talbot

**Affiliations:** 1 Cell Molecular and Developmental Biology Graduate Program, University of California, Riverside, CA 92521 United States; 2 UCR Stem Cell Center, University of California, Riverside, CA 92521 United States; 3 Department of Molecular, Cell and Systems Biology, University of California, Riverside, CA 92521 United States; 4 Department of Civil and Environmental Engineering, Portland State University, PO Box 751, Portland, OR 97207-0751 United States

## Abstract

Thousands of electronic cigarette refill fluids are commercially available. The concentrations of nicotine and the solvents, but not the flavor chemicals, are often disclosed on product labels. The purpose of this study was to identify and quantify flavor chemicals in 39 commercial refill fluids that were previously evaluated for toxicity. Twelve flavor chemicals were identified with concentrations ≥1 mg/ml: cinnamaldehyde, menthol, benzyl alcohol, vanillin, eugenol, *p*-anisaldehyde, ethyl cinnamate, maltol, ethyl maltol, triacetin, benzaldehyde, and menthone. Transfer of these flavor chemicals into aerosols made at 3V and 5V was efficient (mean transfer = 98%). We produced lab-made refill fluids containing authentic standards of each flavor chemical and analyzed the toxicity of their aerosols produced at 3V and 5V using a tank Box Mod device. Over 50% of the refill fluids in our sample contained high concentrations of flavor chemicals that transferred efficiently to aerosols at concentrations that produce cytotoxicity. When tested with two types of human lung cells, the aerosols made at 5V were generally more toxic than those made at 3V. These data will be valuable for consumers, physicians, public health officials, and regulatory agencies when discussing potential health concerns relating to flavor chemicals in electronic cigarette products.

## Introduction

In 2014, more than 7,000 electronic cigarette (EC) refill fluid products with unique flavor names were commercially available online and from local shops, and the number of products continues to grow^1^. In a study of 30 refill fluids, total flavor chemical concentrations ranged from 10–40 mg/ml^2^. Even though many of these flavor chemicals are generally regarded as safe (GRAS) for ingestion, the Flavors & Extract Manufacturers Association (FEMA) has cautioned that their GRAS designation does not extend to inhalation^3^. It is important to gain a better understanding of which flavor chemicals are predominant ingredients in refill fluids, their concentrations, and the potential health effects that EC consumers may experience when inhaling high concentrations of flavor chemicals.

As an initial step to identify refill fluids that may have adverse health effects, our laboratory evaluated the cytotoxicity of a library of refill fluids with a broad range of flavors which included buttery/creamy, minty, sweet/candy, fruit, tobacco and cinnamon/spiced^4^. About a third of these products were highly cytotoxic to human pulmonary fibroblasts (hPF) and two types of stem cells. However, a cinnamon-flavored refill fluid was the most potent across all cell types. Cinnamaldehyde was subsequently identified as the dominant flavor chemical in a small library of commercial cinnamon-flavored refill fluids, and its concentration was directly correlated with its cytotoxicity in the MTT assay^5^.

The cytotoxicity of the refill fluids and aerosols made from the products in our original screen^4^ was then compared across brands^6^. 74% of the refill fluid cytotoxicity data accurately predicted the toxicity of the corresponding aerosols. The “creamy/buttery”-flavored refill fluids led to more cytotoxic aerosols than did fluids in other flavor classes, again suggesting that flavor chemicals are differentially important in determining cytotoxicity of EC products.

The above studies have demonstrated a need for further identification and characterization of the potency of flavor chemicals in EC refill fluids. The current study evaluated the flavor chemicals and their cytotoxicity using the two refill fluid libraries from our previous studies^4,5^, which included 39 commercial refill fluids and six duplicate bottles (45 products total). We specifically: (1) identified the flavor chemicals in each product, (2) produced “lab-made” refill fluids with authentic standards of those flavor chemicals present in concentrations ≥1 mg/ml, (3) determined how much of each flavor chemical is transferred from the lab-made refill fluids into aerosols made at high and low voltages, (4) examined the cytotoxicity of these aerosols using human lung cells, and (5) identified reaction products formed due to the aerosolization process.

## Results

### Identification of flavor chemicals in 39 refill fluids and selected duplicates

**Figure 1:**
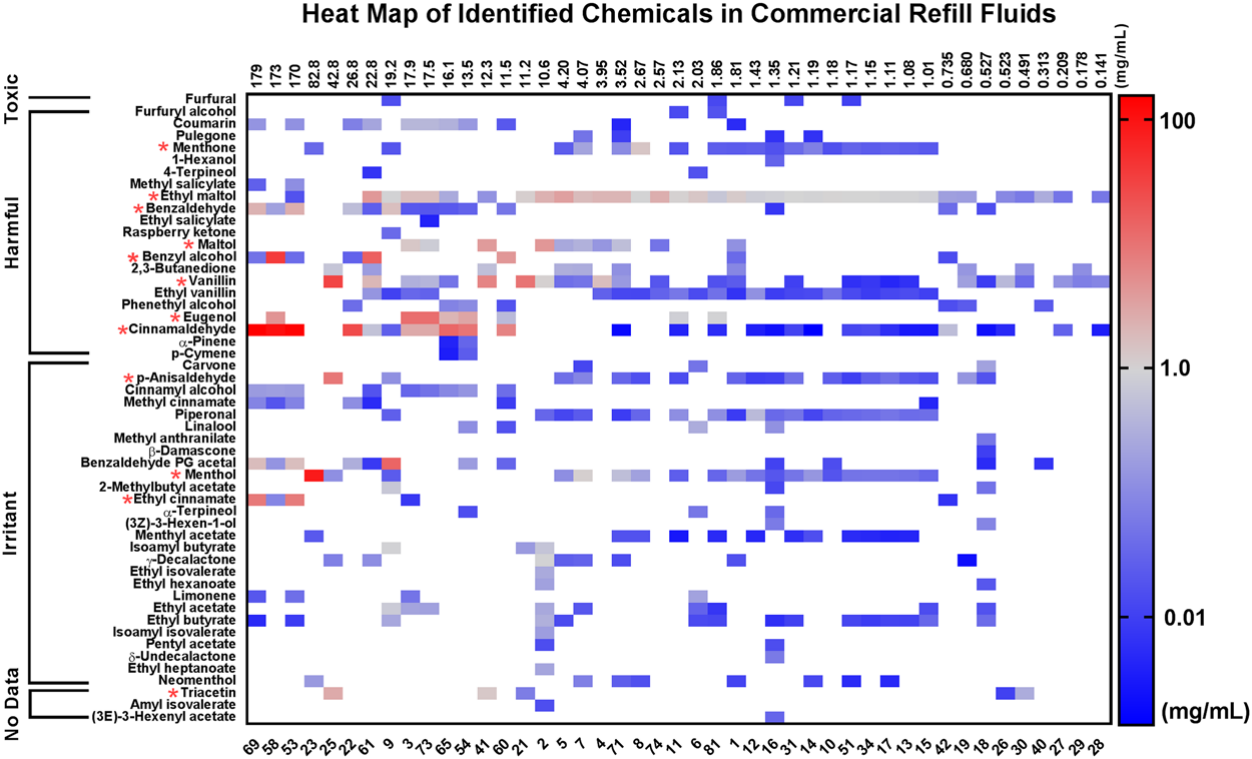
Heat map of flavor chemicals identified and quantified in 45 commercial refill fluids. Chemicals (*y*-axis) are classified by toxicity and ordered within each toxicity bracket from highest to lowest lethal dose based on rat oral toxicity data. Refill fluids (*x*-axis) are represented by their inventory number and ranked with the left having the highest concentration of total flavor chemicals and the right having the lowest. The color gradient provides information on the concentration of each chemical. Red asterisks represent the 12 flavor chemicals that were ≥1 mg/ml in at least one product.

Fifty-two of the 92 flavor chemicals on the GC/MS target analyte list used were identified and quantified in the combined library of 45 refill fluids (Fig. 1)^4,5^. The total concentration of the flavor chemicals in these products ranged from 0.141 to 179 mg/ml. The chemicals in Fig. 1 are arranged on the *y*-axis based on IC_50_ (highest to lowest) from rat oral toxicity values found in online compilations^7^. Products on the *x*-axis are identified by our laboratory inventory number (Supplemental Table 1) and are arranged from highest (left) to lowest total flavor chemical concentration. Thirteen chemicals were identified as dominant flavor chemicals, being found at concentrations ≥1 mg/ml in at least one of the products and are identified in Fig. 1 with a red asterisk. The 13^th^ dominant flavor chemical, benzaldehyde PG (propylene glycol) acetal (13.8 mg/ml) was not available commercially for use as an authentic standard and was not examined in this work.

When arranged by toxicity classification according to rat oral data^7^ (*y-*axis of Fig. 1), eight of these 12 flavor chemicals were in the harmful bracket, three were in the irritant bracket, and one was not classified according to oral rat toxicity data^7^ (Fig. 1). These dominant flavor chemicals include mainly minty, sweet, fruity, and creamy flavors (Table 1). The highest concentrations for the 12 dominant flavor chemicals found in the refill fluids were: cinnamaldehyde (155 mg/ml), menthol (84 mg/ml), benzyl alcohol (39 mg/ml), vanillin (31 mg/ml), eugenol (12 mg/ml), *p*-anisaldehyde (9.0 mg/ml), ethyl cinnamate (8.5 mg/ml), maltol (4.9 mg/ml), ethyl maltol (4.2 mg/ml), triacetin (2.8 mg/ml), benzaldehyde (2.5 mg/ml), and menthone (1.4 mg/ml).

**Table 1:** Chemical and cytotoxicity information for the 12 dominant flavor chemicals and the 80% propylene glycol blank.

Chemical Name	CAS #	Flavor Category	Identified Conc. (mg/ml)	A549 <70% of CN		hPF <70% of CN	
				3V	5V	3V	5V
Cinnamaldehyde	14371-10-9	Cinnamon/Spicy	155	Yes	Yes	Yes	Yes
Menthol	2216-51-5	Minty/Cooling/Fresh	84	No	Yes	Yes	Yes
Benzyl Alcohol	100-51-6	Fruity	39	Yes	Yes	Yes	Yes
Vanillin	121-33-5	Vanilla/Sweet/Creamy	31	No	Yes	Yes	Yes
Eugenol	97-53-0	Sweet/Warm Spiced	12	No	Yes	Yes	Yes
*p*-Anisaldehyde	123-11-5	Creamy/Powdery/Vanilla	9.0	No	Yes	No	Yes
Ethyl Cinnamate	103-36-6	Balsamic/Fruity/Berry	8.5	No	Yes	Yes	Yes
Maltol	118-71-8	Sweet/Cotton Candy/Caramellic	4.9	No	Yes	Yes	Yes
Ethyl Maltol	4940-11-8	Sweet/Cotton Candy/Caramellic	4.2	No	Yes	Yes	Yes
Triacetin	102-76-1	Creamy	2.8	No	Yes	No	Yes
Benzaldehyde	100-52-7	Sweet/Cherry/Almond	2.5	No	Yes	No	Yes
Menthone	14073-97-3	Minty/Cooling/Fresh	1.4	No	Yes	No	No
Propylene Glycol	57-55-6	Sweet	—	No	Yes	No	Yes

Identified Conc. represents the highest concentration above 1 mg/ml for each chemical detected in the commercial refill fluid screen using GC/MS. <70% of CN denotes a cytotoxic response in the MTT (3-(4,5-dimethylthiazol-2-yl)-2,5-diphenyltetrazolium bromide) assay that fell below 70% of the control.

### Frequency of occurrence of the 12 dominant chemicals

**Figure 2:**
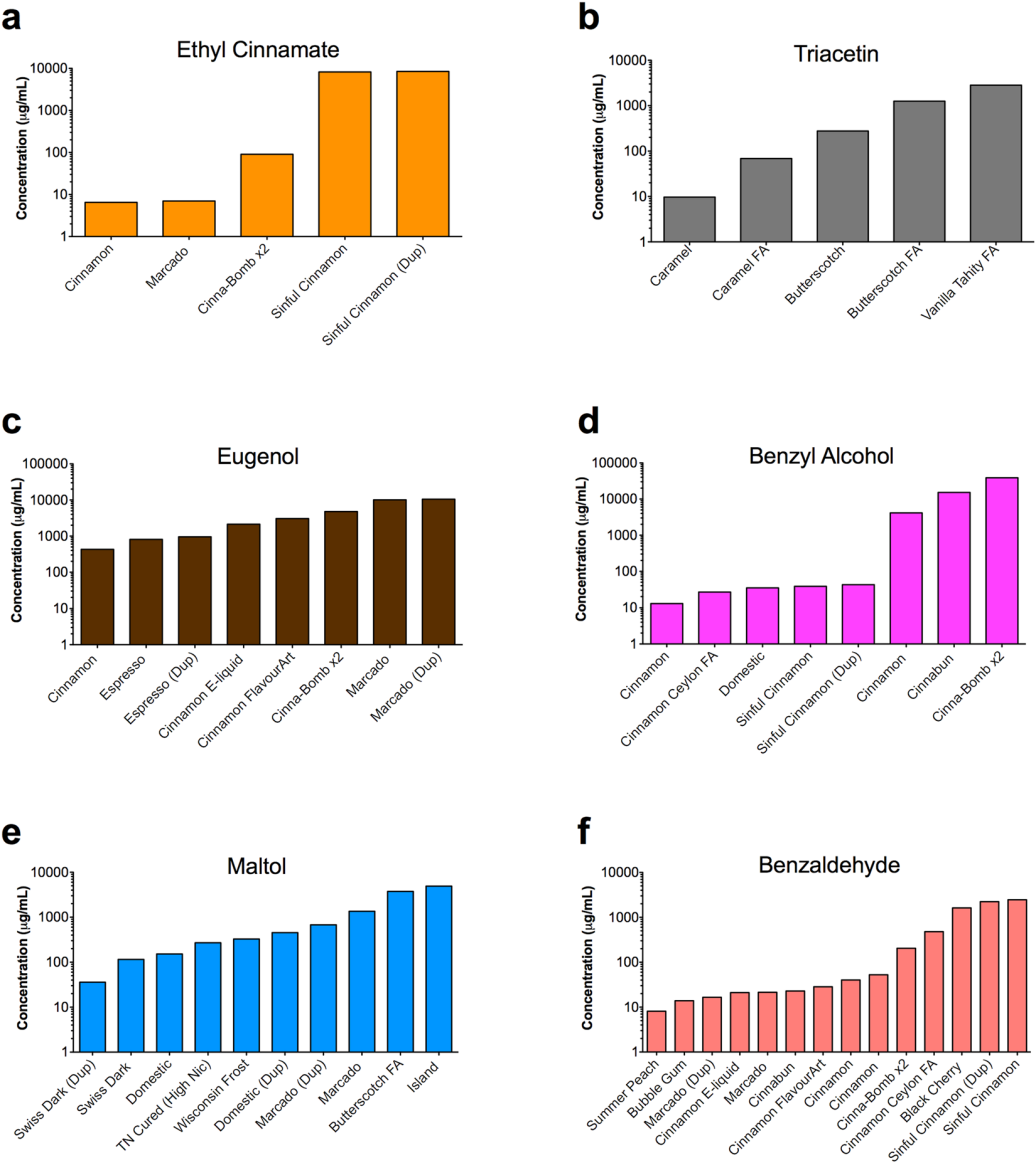
Flavor chemicals present at ≥1 mg/ml that were in <35% of the refill fluids. Duplicate bottles were not incorporated into the frequency calculation, but rather added to distinguish whether duplicate products were similar. (**a**) Ethyl cinnamate was in four products (10%). (**b**) Triacetin was in five products (12.8%). (**c**) Eugenol was in six products (15.4%). (**d**) Benzyl alcohol was in seven products (17.9%). (**e**) Maltol was in seven products (17.9%). (**f**) Benzaldehyde was in 12 products (30.75%). Dup = duplicate bottle purchased and screened.

**Figure 3:**
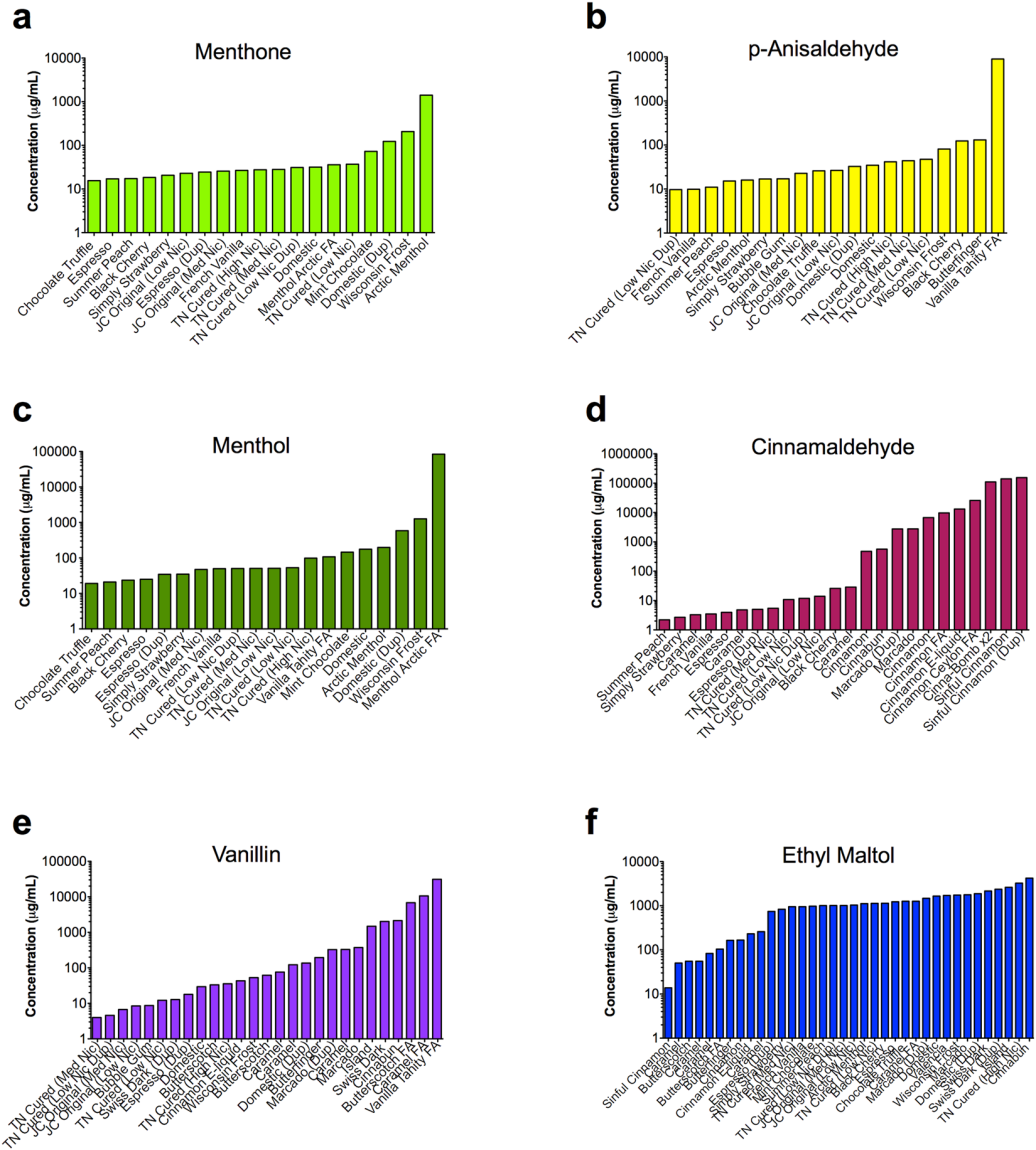
Flavor chemicals present at ≥1 mg/ml that were in >35% of the 39 refill fluids. Duplicate bottles were not incorporated into the frequency calculation, but rather added to distinguish whether duplicate products were similar. (**a**) Menthone was in 16 products (41%). (**b**) *p*-Anisaldehyde was in 17 products (43.6%). (**c**) Menthol was in 17 products (43.6%). (**d**) Cinnamaldehyde was in 20 products (51.2%). (**e**) Vanillin was in 22 products (56.4%). (**f**) Ethyl maltol was in 31 products (79.5%). Dup = duplicate bottle purchased and screened.

The frequency of occurrence and the concentration of each dominant flavor chemical from the GC/MS commercial refill fluid screen is shown in Figs 2 and 3. Duplicate bottles were not incorporated into the frequency calculation, but were included in the graphs to determine whether duplicate products were similar to each other. Six of the 12 dominant flavor chemicals were present less frequently (5–12 products) (Fig. 2). This included ethyl cinnamate in four products (10%), triacetin in five products (13%), eugenol in six products (15%), benzyl alcohol in seven products (18%), maltol in seven products (18%), and benzaldehyde in 12 products (31%). The other six dominant flavor chemicals were found more frequently (16–31 products) (Fig. 3). Figure 3 shows menthone in 16 products (41%), *p*-anisaldehyde in 17 products (44%), menthol in 17 products (44%), cinnamaldehyde in 20 products (51%), vanillin in 22 products (56%), and ethyl maltol in 31 products (80%). Generally, for Figs 2 and 3, the concentrations of flavor chemicals were similar in the duplicate bottles.

### Flavor chemical aerosolization transfer efficiency

**Figure 4:**
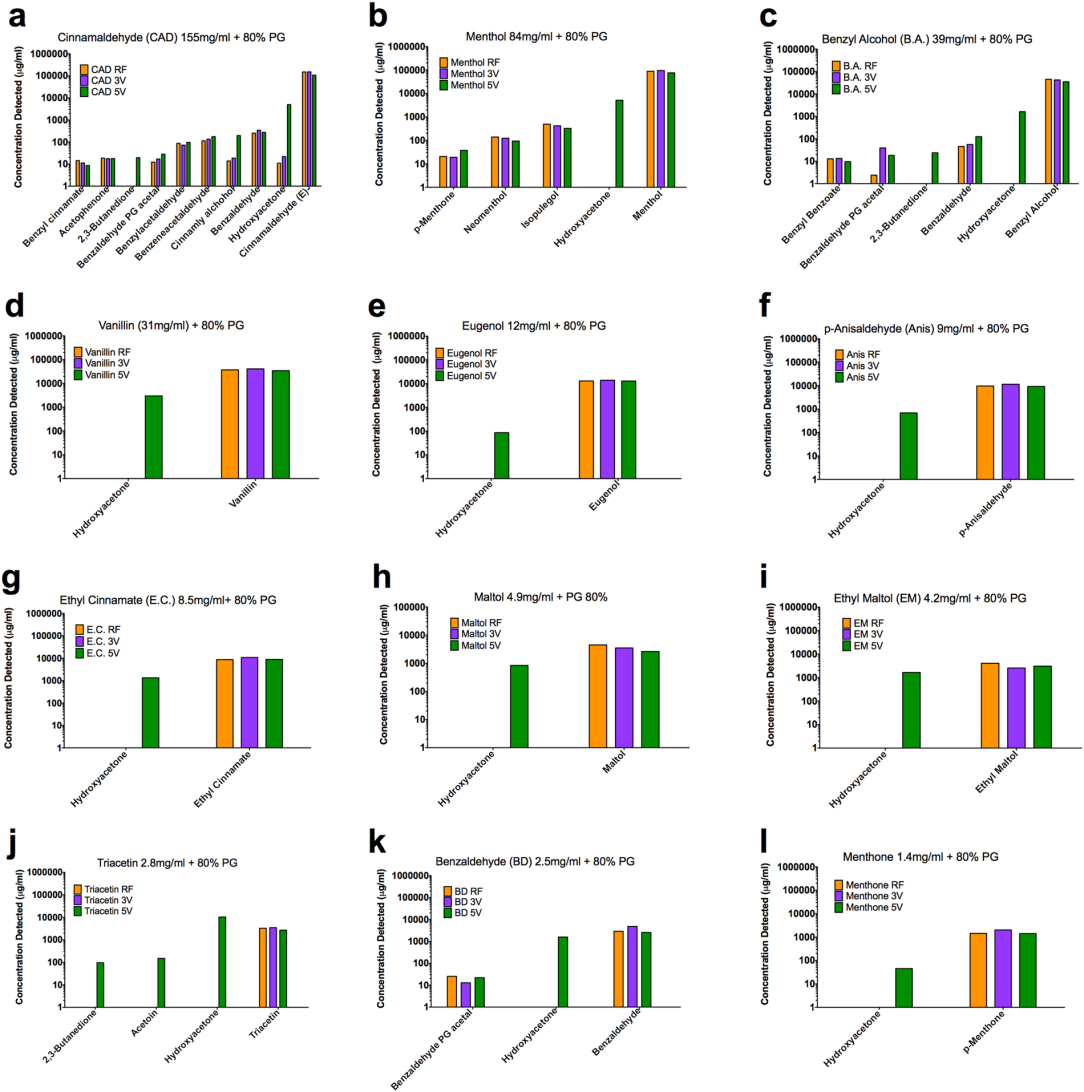
Chemicals in lab-made refill fluids and their corresponding aerosols. (**a**) Cinnamaldehyde. (**b**) Menthol. (**c**) Benzyl alcohol. (**d**) Vanillin. (**e**) Eugenol. (**f**) *p*-Anisaldehyde. (**g**) Ethyl Cinnamate. (**h**) Maltol. (**i**) Ethyl maltol. (**j)** Triacetin. (**k**) Benzaldehyde. (**l**) Menthone. PG = propylene glycol. RF = refill fluid. 3V = 3V aerosol. 5V = 5V aerosol.

Aerosols that were captured in liquids and used for analytical chemistry or cytotoxicity experiments will be referred to as “aerosols”. GC/MS was used to identify and quantify flavor chemicals in aerosols produced at 3V (volts)/4.3 watts or 5V/11.9 watts from lab-made refill fluids that contained 80% propylene glycol and one of the 12 dominant flavor chemicals. For these analyses, the GC/MS target list included 178 flavor chemicals. Generally, for aerosols produced at 3V and 5V, the refill fluid to aerosol transfer efficiency was high (Fig. 4 and Table 2). The 3V aerosolization process had a transfer efficiency of ~80% or greater (except for ethyl maltol which was 62%) and an average transfer efficiency of 110% ± 8%, which was not significantly different than a theoretical mean of 100% (Table 2). Aerosolization at 5V had a transfer efficiency of ~70% or greater (with exception of maltol which was 58%) and an average transfer efficiency of 86% ± 4% which was significantly different than 100% (Table 2).

**Table 2:** Transfer efficiency of each dominant flavor chemical from refill fluid into aerosol.

Chemical Name	3V Aerosol (%)	5V Aerosol (%)
Ethyl Maltol	62	75
Maltol	78	58
Benzyl Alcohol	94	77
Cinnamaldehyde	100	71
Triacetin	106	82
Menthol	107	86
Eugenol	108	99
Vanillin	109	93
*p*-Anisaldehyde	119	96
Ethyl Cinnamate	124	102
Menthone	141	99
Benzaldehyde	168	88
Average ± SEM	110 ± 8	86 ± 4**

Chemicals are ordered from lowest to highest transfer efficiency in the 3V column. SEM = standard error of the mean. The asterisks indicate that the average transfer efficiency is statistically significant when compared to a theoretical mean of 100%. **p < 0.01.

### Identification of co-constituents and secondary reaction products

The refill fluids and aerosols made from the three dominant chemicals with the highest concentrations (cinnamaldehyde, menthol, benzyl alcohol) contained several chemicals at low concentration that likely were co-constituents of the dominant flavor chemical (Fig. 4a–c). To determine if new chemicals were produced upon aerosolization, refill fluids were compared to aerosols collected at 3V and 5V. No new chemicals from our target analyte list were found at significant concentrations for the 3V aerosol. Three new chemicals were present in aerosol made at 5V. Hydroxyacetone appeared in all 5V aerosols at concentrations ranging from ~100 to ~10,000 µg/mL (Fig. 4). Acetoin was present at 150 µg/mL in the 5V aerosol of triacetin (Fig. 4j), and 2,3-butanedione (diacetyl) was present in the 5V aerosol of cinnamaldehyde, benzyl alcohol, and triacetin at 20, 24 and 96 µg/mL, respectively.

### Cytotoxicity of hydroxyacetone

**Figure 5:**
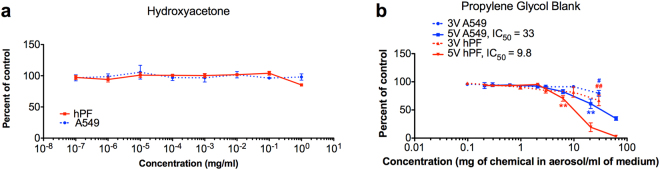
Dose-response curves for hydroxyacetone and 80% propylene glycol blank. (**a**) Hydroxyacetone diluted directly into culture medium for A549 cells and hPF. (**b**) A549 cells and hPF treated with 80% propylene glycol aerosolized at 3V and 5V and captured in culture medium. IC_50_ represents the concentration that inhibited survival by 50% and is only indicated where applicable. Data are showing the means and their standard errors for three independent experiments. Red lines represent hPF dose-response curves. Blue lines represent A549 dose-response curves. Hash symbols (#) for 3V aerosols and asterisks (*) for 5V aerosols indicate the lowest concentration that is significantly different than the untreated controls. *p < 0.05, **p < 0.01.

Because hydroxyacetone appeared in all 5V aerosols, its cytotoxicity was evaluated using the MTT assay with hPF and the A549 CCL-185 lung epithelial cell line (A549) (Fig. 5a). Even at 1 mg/ml (1.35 × 10^−2^ M), hydroxyacetone did not inhibit metabolic activity of hPF or A549 cells, and it was not deemed cytotoxic for either cell type using ISO 10993-5:2009(E) international standard^8^, which categorizes cytotoxicity based on a treatment producing an absorbance in the MTT assay that is <70% of the untreated control.

### Cytotoxicity of aerosols made from propylene glycol and lab-made refill fluids

An 80% propylene glycol/20% water blank aerosolized at 5V (but not at 3V) produced IC_50_s for both cell types (Fig. 5b). Using the cytotoxicity categorization from ISO 10993-5, 5V samples were cytotoxic and had a response that fell below 70% of control in the MTT assay, while 3V samples were not cytotoxic for either cell type (Table 1), in agreement with our previous study^6^.

**Figure 6:**
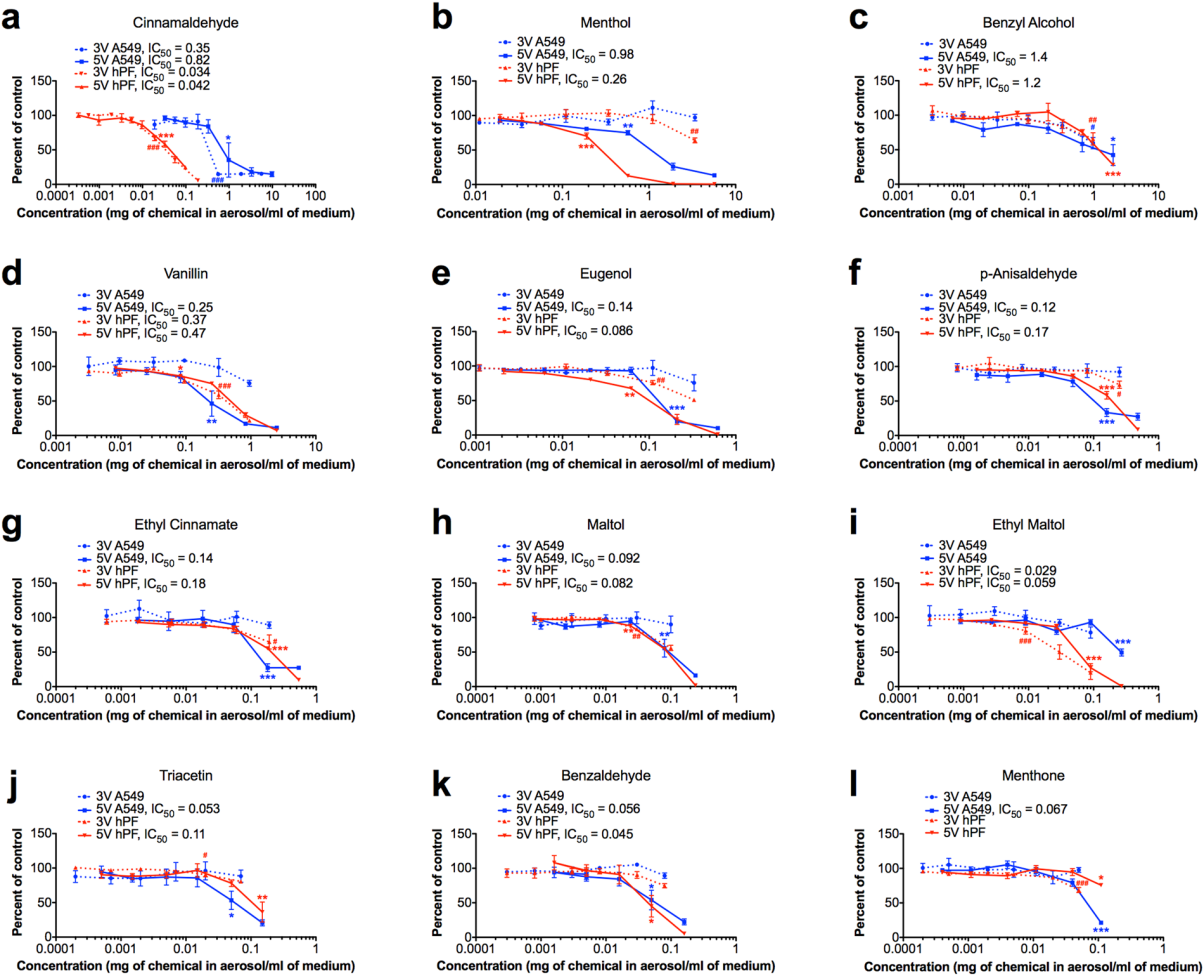
Dose-response curves for A549 cells and hPF treated with aerosols produced from lab-made refill fluids containing the dominant flavor chemicals. The *y-*axis shows percent of survival in the MTT assay and is normalized to the untreated controls. The *x*-axis shows the mg of chemical per mL of culture medium. (**a–l**) represents each of the 12 dominant chemicals, prepared in refill fluid form, then collected as an aerosol into culture medium. IC_50_ represents the inhibitory concentration at 50% and is only indicated where applicable. Data are showing the means and their standard errors for three independent experiments. Red lines represent hPF dose-response curves. Blue lines represent A549 dose-response curves. Hash symbols (#) for 3V aerosols and asterisks (*) for 5V aerosols indicate the lowest concentration that is significantly different from the untreated controls. *p < 0.05, **p < 0.01, ***p < 0.001.

A549 cells and hPF were exposed to 3V and 5V aerosols from lab-made refill fluids that contained one dominant flavor chemical at its highest identified concentration (Table 1) in 80% propylene glycol and ~20% water (Fig. 6). Generally, aerosols generated at 5V were more potent than those made at 3V (Fig. 6). Exceptions to this were aerosols created from refill fluids containing cinnamaldehyde (at 155 mg/mL), vanillin (hPF) (at 31 mg/mL), and ethyl maltol (hPF) (at 4.2 mg/mL), for each of which the 3V and 5V aerosols produced similar results (Fig. 6). When comparing sensitivity in the MTT assay using ISO 10993-5, 67% of the 3V aerosols and 92% of the 5V aerosols were cytotoxic to hPF, while 17% of the 3V aerosols and 100% 5V aerosol samples were cytotoxic to A549 (Table 1). The hPF were considerably more sensitive to cinnamaldehyde and menthol than the A549 cells.

## Discussion

Relatively little is known about the flavor chemicals used in EC refill fluids and their effects on human health. In this study, we identified and quantified flavor chemicals in 39 commercial refill fluids and evaluated their cytotoxicity. Because nicotine concentration and fluid color vary in duplicate bottles of refill fluids^9^, we also compared six pairs of duplicate products, and in each case the duplicate bottle was generally similar to the original bottle’s flavor chemical profile. Total flavor chemical concentrations in these products ranged from 0.141 to 179 mg/ml with 29% of the products having concentrations >12 mg/ml, which is higher than the median nicotine concentration preferred by EC consumers^10,11^. At least one of the 12 dominant flavor chemicals (i.e., those that were ≥1 mg/ml) was present in 56% of the original 39 products. Some chemicals were used frequently. For example, menthone, *p*-anisaldehyde, menthol, cinnamaldehyde, vanillin, and ethyl maltol appeared in 41–80% of the refill fluids in our library. Our data further showed that the flavor chemicals in lab-made refill fluids transferred efficiently into the aerosol. These data support the conclusion that flavor chemicals are a major component of EC refill fluids and that many commercial refill fluids would expose EC users to high concentrations of certain flavor chemicals.

The cytotoxicity of the products screened by Bahl *et al*.^4^ generally correlated with the concentration of a specific flavor chemical(s) or the total concentration of flavor chemicals. In Supplemental Table 2, the IC_50_ data from the initial refill fluid screen on hPF^4^, in which the highest dose tested was 1%, is compared to the total concentration of flavor chemicals and the individual dominant flavor chemicals that were in each product. Of the nine highly cytotoxic refill fluids in the screen by Bahl *et al*.^4^, six had total flavor chemical concentrations >11 mg/ml (#3, 23, 41, 21, 25, 22) and seven (#3, 23, 4, 41, 21, 25, 22) contained one or more dominant flavor chemical at a concentration that would have been cytotoxic in the original screen. It is possible that the remaining two refill fluids (#12 and #40) also contained cytotoxic flavor chemicals that were not present on our GC/MS target analyte list, although we cannot rule out the possibility that toxicity was due to a non-flavor chemical, such as a metal^12^.

Most refill fluids that had low cytotoxicity had total flavor chemical concentrations <5 mg/ml, which distinguishes them from the highly cytotoxic group and supports our hypothesis that toxicity in the original screen was due to high concentrations of flavor chemicals. For those refill fluids that contained one or more dominant flavor chemicals and had high total flavor concentrations but were not cytotoxic in the original screen (#1, 2, 5, 6, 7, 8, 9, 10 and 11), it is possible that the mixture of chemicals in the refill fluids decreased cytotoxicity in the MTT assay or that their individual concentrations were not high enough to be cytotoxic (most were <3 mg/ml).

The transfer efficiency data demonstrate that aerosols inhaled by EC consumers can contain high concentrations of flavor chemicals that in many cases equaled the concentrations in the parent refill fluids. Additionally, even flavor chemicals present in low concentrations in fluids also transferred efficiently to the aerosols, in agreement with a previous study on diacetyl and acetyl propionyl in propylene glycol/glycerol^13^. Overall, the 5V aerosol samples had a lower transfer efficiency than the 3V samples. While the reason for this is not currently known, it is possible that higher temperatures at 5V oxidized the flavor chemicals thereby reducing their transfer efficiency or converted them into other chemical reaction products.

To understand the effects of heating the flavor chemicals at a high voltage, the cytotoxic impact of compounds formed directly from the solvent at 5V were evaluated. Hydroxyacetone, which was identified in every 5V sample, forms from the breakdown of propylene glycol^14,15^. Hydroxyacetone was not cytotoxic to hPF or A549 cells in the MTT assay, but could be effective in other cytotoxicity assays. Additionally, carbonyl compounds, such as formaldehyde and acrolein, form when the solvents in refill fluids are heated at high voltages^16–18^, and these could have contributed to the greater toxicity seen in the 5V samples.

Additional secondary reaction products generated from 5V samples included 2,3-butanedione (diacetyl) (~20–100 µg/ml), which was identified in cinnamaldehyde, benzyl alcohol and triacetin, and acetoin (150 µg/ml), which was identified in triacetin. The origin of these secondary products was not determined, but could be the flavor chemicals and/or the solvents. Other studies have observed diacetyl and acetoin in refill fluids and/or aerosols^13,18–20^. Acetoin has a similar chemical structure as well as organoleptic and physical properties to 2,3-butanedione, which can cause obliterative bronchiolitis and affects epithelial barrier function^21,22^. The National Institute for Occupational Safety and Health (NIOSH) recommends that exposure to 2,3-butanedione be limited to 5 parts per billion for up to 8 hours/day during a 40-hour work week. Farsalinos *et al*.^13^ interpreted the NIOSH recommended safety limits of 2,3-butanedione in reference to ECs and defined acceptable daily inhalation limit as 65 µg^13^. In our study, the 5V aerosol sample for triacetin produced 96 µg/ml of 2,3-butanedione from 60 puffs, which is equivalent to 1.6 µg/puff. Taking 1.6 µg/puff and multiplying it with the mean puffs/day value that was averaged across three independent studies (155 puffs/day)^23–25^ equals 248 µg of 2,3-butanedione/day. This level is almost four times higher than the extrapolated safety limit. Individuals using ECs at high voltages/wattages should be aware that 2,3-butanedione can be produced at potentially dangerous levels by heating refill fluids. Moreover, when attempting to establish toxicity of a flavor chemical versus its reaction product(s), it would be advisable to generate aerosols at 3V or a lower voltage to minimize forming secondary products from the solvent and/or flavor chemical.

The 12 dominant chemicals were also present in lower concentrations (<1 mg/ml) in some of the commercial refill fluid products. The chemicals present in extremely low concentrations (≤10 µg/ml) are likely co-constituents of the dominant flavor chemicals. Even authentic standards with high purity can introduce low concentrations of co-constituent chemicals (sometimes ≥10 µg/ml), as seen with our lab-made refill fluids containing cinnamaldehyde, menthol, benzyl alcohol and benzaldehyde.

Recent studies have determined the concentrations of select flavor chemicals in purchased refill fluids (cinnamaldehyde^20,26^, diacetyl & 2,3-pentanedione^13,19^ and benzaldehyde^27^), and have addressed the chemical profiles or major flavor compounds in commercial products^2,28–30^. In our study, the dominant flavor chemicals were not only identified and quantified, but also tested for cytotoxicity using the highest concentration observed in the commercial products. The aerosols derived from these high concentration lab-made refill fluids varied in cytotoxicity (Table 1) and potency (Fig. 6) across both lung cell types. The variation between samples verifies that the cytotoxic responses seen in different samples are not due to propylene glycol. Although propylene glycol was cytotoxic at 5V, the dose-response curves for the aerosols were not all similar to the propylene glycol aerosols for either cell type. Moreover, unlike propylene glycol, flavor chemical aerosols were cytotoxic at 3V, especially with hPF. Flavor chemicals that were potent and found frequently include ethyl maltol and vanillin, which were both cytotoxic to hPF (3V and 5V aerosols) and A549 cells (5V only) and found in over 50% of the products. In agreement with our study, ethyl maltol was cytotoxic and vanillin induced physiological alterations to human bronchial epithelial cells^31^.

hPF were often more sensitive to aerosols than the A549 cells at 3V (Table 1 and Supplemental Table 3), while at 5V, hPF and A549 cells were equally effected (Table 1 and Supplemental Table 3). For hPF, 3V and 5V aerosols from refill fluids containing cinnamaldehyde, ethyl maltol, and vanillin generated similar dose-response curves (Fig. 6), suggesting that the flavor chemicals alone caused the response, which was not increased by generation of reaction products at 5V. This distinguishes hPF as the more sensitive cell type, in agreement with our previous work^20^. The A549 cells are a carcinoma-derived line that is generally more robust than primary cells^32^. This difference in sensitivity is seen in the dose-response curves for propylene glycol, cinnamaldehyde, menthol, and ethyl maltol (Figs 5 and 6) and the cytotoxicity assessment in Table 1.

In conclusion, our study shows that: (1) in a screen of 39 commercial refill fluids, 12 dominant flavor chemicals (≥1 mg/ml) were identified in 56% of the products; (2) flavor chemical profiles of each product differ with total flavor chemical concentrations ranging from 0.141 to 179 mg/ml; (3) the chemicals varied in frequency of occurrence with vanillin and ethyl maltol found in over 50% of the products; (4) flavor chemicals and their concentrations were similar in duplicate bottles of six different products; (5) 3V and 5V aerosols often had concentrations of flavor chemicals similar to those in the parent lab-made fluids; (6) high concentrations of authentic standards of flavor chemicals introduce co-constituents, which transfer to the aerosols; (7) no reaction products were identified in 3V aerosols; (8) hydroxyacetone, a product of heating propylene glycol, was identified in all 5V aerosols; (9) hydroxyacetone was not cytotoxic to hPF or A549 cells in the MTT assay; (10) the 80% propylene glycol blank was cytotoxic to both cell types at 5V; (11) the dominant flavor chemicals were more cytotoxic than the propylene glycol blank; (12) 5V samples were generally more potent than 3V samples to both cell types; (13) there was clear evidence of secondary reaction product formation in aerosols produced at 5V; (14) 2,3-butanedione (in cinnamaldehyde, benzyl alcohol, and triacetin) and acetoin (in triacetin) were found in 5V samples and present health concerns; (15) overall, hPF were more sensitive to the aerosols generated from lab-made refill fluids than A549 cells; (16) in general, the cytotoxicity first observed for the refill fluids by Bahl *et al*.^4^ can be attributed to high concentrations of flavor chemicals in some of the fluids. This study offers new information on the identification, concentration and cytotoxicity of flavor chemicals in EC refill fluids that will help advance research in this area, guide the regulation of EC products (e.g., by limiting concentration of all or specific flavor chemicals), and assist medical professionals treating EC consumers.

## Materials and Methods

### Refill fluids and authentic standards

Thirty-nine commercial EC refill fluids and duplicate bottles of six of these products (45 total products) were purchased from online vendors including Freedom Smoke USA (Tucson, AZ), Global Smoke (Los Angeles, CA), Johnson Creek (Johnson Creek, WI), Red Oak (a subsidiary of Johnson Creek), Tasty Puff (Albuquerque, NM), e-cigexpress (Orlando, FL), Vaporbomb.com (Barberton, OH), Vapormaxx (Richmond, VA), and DIY Flavor Shack (Las Vegas, NV). Inventory number, flavor name, and company are given in Supplemental Table 1 and details of each product have been previously published^4–6^. Commercial fluids were stored in 4 °C and cytotoxicity did not change with storage^6^.

Authentic standards of chemicals: (1) trans-cinnamaldehyde from TCI (Tokyo, Japan), (2) L-menthone and hydroxyacetone from Alfa Aesar (Ward Hill, MA, USA), (3) ethyl maltol, L-menthol, maltol, benzyl alcohol, ethyl cinnamate, eugenol, *p*-anisaldehyde, triacetin, vanillin, and benzaldehyde from Sigma-Aldrich (St. Louis, MO, USA), and (4) propylene glycol from Acros Organics (New Jersey, USA). Triacetin is a flavor chemical as well as a solvent. Each authentic standard was dissolved in propylene glycol (80%) and distilled water (<20%) to simulate hand-made refill fluids. A propylene glycol control blank was prepared with 80% propylene glycol and 20% distilled water.

### Identification and quantification of flavor chemicals in commercial refill fluids, lab-made fluids, and aerosols using GC/MS

Commercial refill fluids were analyzed by GC/MS. Internal standard-based calibration procedures similar to those described elsewhere^2^ were used, and analyses for 92 flavor-related target analytes were performed with an Agilent (Santa Clara, California, USA) 7693 autosampler, Agilent 7890A GC and Agilent 5975C MS. The capillary column used was of type DB-VRX (60 m × 250 µm × 1.4 µm film thickness). The relatively high film thickness was chosen to provide the column with adequate capacity for compounds present at high concentrations. For each refill fluid sample, 50 µL was dissolved in 950 µL of isopropanol (Fisher Scientific, Fair Lawn, New Jersey, USA); 20 µL of internal standard solution (2 µg/µL of 1,2,3-trichlorobenzene in isopropyl alcohol) was added before analysis; 1 µL was injected into the GC/MS with a 10:1 split. The GC temperature program was: 45 °C hold for 5 min; 12 °C/min to 189 °C; hold at 189 °C for 2 min; 5 °C/min to 245 °C, then hold for 10 min at 245 °C. The MS was operated in electron impact (EI) ionization mode and positive ion detection mode. The ion source temperature was 220 °C and the quadrapole temperature was 150 °C. The scan range was from 34 to 400amu. Each target analyte was quantitated using: a) authentic standard material; b) its specific quantitation ion; and c) internal-standard (1,2,3-trichlorobenzene)-normalized multipoint calibration based on peak area.

For analyses of lab-made fluids and aerosols produced by vaping at 3V and 5V, all analytical conditions were the same except as follows: a) the target analyte list was expanded to 178 flavor-related compounds; b) the GC column was from Restek (Bellefonte, PA), and of type Rxi-624Sil MS (30 m × 250 µm × 1.4 µm); c) the GC temperature program was: 40 °C hold for 2 min; 10 °C/min to 100 °C; 12 °C/min to 280 °C; hold for 8 min at 280 °C; then 10 °C/min to 230 °C. Concentrations of the chemicals reported for the collected liquid aerosol material were made by: a) weighing the EC device before and after vaping to determine the mass of aerosolized EC fluid; b) assuming exactly that mass was collected during aerosol sample collection; and c) accounting for the dilution with isopropanol in the aerosol sampling device.

### EC aerosol production and capture using an impinger method

Aerosols were produced with two highly rated and popular EC components: an Innokin iTaste MVP 3.0 battery with variable voltage (3V–9V) and wattage (6–30 watts) and fresh unused Innokin iClear16D dual coil clearomizers (or tanks) using a smoking machine that contained a Cole-Parmer Masterflex L/S peristaltic pump. An atomizer of 2.1 ohms resistance was used to generate aerosols and a voltage of either 3V (4.3 watts) or 5V (11.9 watts) was applied. Clearomizers were loaded with 2 ml of lab-made refill fluid and were not allowed to drop below 0.5 mL of fluid in order to avoid dry puffing. Puff duration was 4.3 sec, the average for EC consumers^33^, and a flow rate of 13 mL/sec was used to produce 56 mL puffs every 60 sec.

For analytical chemistry, aerosols were collected at room temperature in two 125 mL impingers, each containing 25 mL of isopropanol. Following the large impingers was a third 30 mL impinger which contained 10 mL of isopropanol to ensure no aerosol was lost. The clearomizer was weighed before and after aerosol production to collect a mass concentration of at least 15 mg/mL for GC/MS analysis. Aerosol solutions were collected, aliquoted, and stored at −20 °C until analyzed.

For cytotoxicity experiments, the aerosol was condensed onto culture medium in a round bottom flask submerged in a dry-ice bath. Weights were taken before and after puffing to monitor fluid consumption. For each batch of aerosol, 6 puffs were collected/mL of culture medium, and aerosol solutions were aliquoted, and stored at −80 °C. Aerosols for cytotoxicity were expressed as total puff equivalents (TPE - the number of puffs/milliliter of culture medium) then later converted into mg of chemical in the aerosol/milliliter of medium. Aerosols were tested at 0.02, 0.06, 0.2, 0.6, 2 and 6 TPE. To convert from TPE to milligrams of the chemical in aerosol/milliliter of medium, the weight difference before and after aerosol collection was utilized. First, take the weight difference, which represents the amount of fluid that was consumed, and divide by the number of milliliters used to collect the aerosol. Then, multiply this value by the percentage of chemical that was in the original lab-made refill fluid. The value is now in the units of mg of chemical in the aerosol/milliliter of medium and is equivalent to the high dose tested.

### Culturing hPF and A549

The hPF (ScienCell, Carlsbad, CA) were cultured on poly-L-lysine coated flasks using the supplier’s protocol in complete fibroblast medium containing 2% fetal bovine serum, 1% fibroblast growth serum, and 1% penicillin/streptomycin. In experiments. hPF were dispersed into single cells and seeded at a density of 4,000 cells/0.32 cm^2^ using a BioMate 3S Spectrophotometer (Thermo Fisher Scientific, Chino, CA) based standard curve.

The A549 cell line (ATCC, Manassas, VA) was cultured using the suppliers protocol in ATCC F-12K medium containing 10% fetal bovine serum on tissue culture flasks. In experiments, cells were collected and seeded as single cells at a density of 50,000 cells/0.32 cm^2^ using a BioMate 3S Spectrophotometer based standard curve.

### Cytotoxicity in the MTT Assay

Chemicals were tested for cytotoxicity using the MTT assay when their concentration was ≥1 mg/ml in at least one refill fluid. Twelve authentic standards of chemicals meeting this criterion were tested in dose response experiments. Each chemical was combined with 80% propylene glycol to produce a lab-made refill fluid for testing. Hydroxyacetone was directly diluted into culture medium with a dose range from 1.0 × 10^−7^ mg/ml to 1 mg/ml. For aerosol samples and hydroxyacetone, cells were seeded in 96-well plates containing control wells, vapor effect control wells (wells adjacent to the highest concentration to ensure vapors did not affect neighboring wells), and treatment wells^34^. MTT reagent dissolved in phosphate buffered saline was added to control and exposed cells after 48 hrs of incubation. After 2 hrs of incubation with MTT solution, formazan crystals were solubilized in dimethyl sulfoxide, and absorbance was read at 570 nm. For each chemical tested, three independent experiments were performed with each cell type. Chemicals were considered cytotoxic when the highest concentration gave a response that was <70% of the control (ISO protocol #10993-5).

### Data analysis

For aerosol dose-response and hydroxyacetone cytotoxicity experiments, the concentrations that inhibited survival by 50% (IC_50_) were computed with GraphPadPrism software (GraphPad, San Diego, California, USA) using the log inhibitor versus normalized response-variable slope with the top and bottom constraints set to 100% and 0%, respectively. Statistical significance for aerosols was determined with GraphPad Prism using an analysis of variance of three independent experiments. When significance was found, treated groups were compared with the lowest concentration using Dunnett’s post hoc test. A one sample t-test with a theoretical mean of 100% was used to compare the average 5V transfer efficiency and the average 3V transfer efficiency to 100%. Means were considered significantly different when p < 0.05.

## Electronic supplementary material

Supplementary Info
