# Supplementary material for: Analytical and toxicological evaluation of flavor chemicals in electronic cigarette refill fluids
Source: Sci Rep. 2018 May 29;8:8288. doi: 10.1038/s41598-018-25575-6 (PMC5974410; doi:10.1038/s41598-018-25575-6)
Supplement: Supplementary file 1 — Supplementary Info [file 41598_2018_25575_MOESM1_ESM.pdf]

Analytical and toxicological evaluation of flavor chemicals in electronic cigarette refill fluids

Rachel Z. Behar, Wentai Luo, Kevin J. McWhirter, James F. Pankow and Prue Talbot

Inventory Number	Refill Fluid	Company
**1**	Domestic	RO
**2**	Island	RO
**3**	Marcado	RO
**4**	Swiss Dark	RO
**5**	Tennessee Cured	RO
**6**	Valencia	RO
**7**	Wisconsin Frost	RO
**8**	Arctic Menthol	JC
**9**	Black Cherry	JC
**10**	Chocolate Truffle	JC
**11**	Espresso	JC
**12**	French Vanilla	JC
**13**	JC Original	JC
**14**	Mint Chocolate	JC
**15**	Simply Strawberry	JC
**16**	Summer Peach	JC
**17**	Tennessee Cured	JC
**18**	Bubblegum	FS
**19**	Butterfinger	FS
**21**	Caramel	FS
**22**	Cinnamon Ceylon	FS
**23**	Menthol Arctic	FS
**25**	Vanilla Tahity	FS
**26**	Caramel	FS
**27**	Caramel	FS
**28**	Caramel	FS
**29**	Butterscotch	FS
**30**	Butterscotch	FS
**31**	Tennessee Cured	JC
**34**	JC Original	JC
**40**	Caramel	GS
**41**	Butterscotch	FS
**71**	Domestic (dup of #1)	RO
**73**	Marcado (dup of #3)	RO
**74**	Swiss Dark (dup of #4)	RO
**81**	Espresso (dup of #11)	JC
**51**	Tennessee Cured (dup of #31)	JC
**42**	Cinnamon	FS
**53**	Sinful Cinnamon	TP
**54**	Cinnamon FlavourArt	FS
**58**	Cinna-Bomb x2	vaporbomb.com
**60**	Cinnamon	e-cigexpress
**61**	Cinnabun	e-cigexpress
**65**	Cinnamon E-Liquid	DIY Flavor Shack
**69**	Sinful Cinnamon (dup of #53)	TP

Supplementary Table 1. Inventory number and commercial refill fluid information. RO = Red Oak. JC = Johnson Creek. FS = Freedom Smoke USA. GS = Global Smoke. TP = Tasty Puff. Dup = Duplicate bottle.

Inv. #	Refill fluid	hPF refill fluid IC_{50} (%) Bahl et al.	Total flavor chemical conc. (mg/ml)	Dominant flavor chemicals? (> 1 mg/ml)
**34**	JC Original	> 1	1.2	-
**27**	Caramel	> 1	0.2	-
**17**	TN cured	> 1	1.1	-
**16**	Summer Peach	> 1	1.3	-
**15**	Simply Strawberry	> 1	1.0	-
**14**	Mint Chocolate	> 1	1.2	-
**13**	JC Original	> 1	1.1	-
**10**	Chocolate Truffle	> 1	1.2	1.03 EM
**8**	Arctic Menthol	> 1	2.7	1.4 Menthone
**6**	Valencia	> 1	2.0	1.4 EM
**9**	Black Cherry	> 1	19.2	1.6 Benzaldehyde, 14 BPGA*****
**18**	Bubblegum	> 1	0.5	-
**11**	Espresso	> 1	2.1	1.1 EM
**31**	TN cured	> 1	1.2	-
**2**	Island	> 1	10.6	1.1 Van, 2.3 EM, 4.9 Maltol
**5**	TN cured	> 1	4.2	3.4 EM
**1**	Domestic	> 1	1.8	1.3 EM
**7**	Wisconsin Frost	> 1	4.1	1.8 EM, 1.1 Menthol
**19**	Butterfinger	> 1	0.7	-
**28**	Caramel	> 1	0.1	-
**30**	Butterscotch	> 1	0.5	-
**29**	Butterscotch	> 1	0.2	-
**26**	Caramel	> 1	0.5	-
**12**	French Vanilla	0.97	1.4	-
**3**	Marcado	0.82	18.0	2.8 CAD, 12 Eug, 1.3 Maltol, 1.7 EM
**23**	Menthol Arctic	0.45	82.8	84 Menthol
**40**	Caramel	0.41	0.3	-
**4**	Swiss Dark	0.30	3.9	2.1 EM, 1.7 Van
**41**	Butterscotch	0.26	12.3	3.8 Maltol, 1.3 Tri, 6.6 Van
**21**	Caramel	0.22	11.2	1.1 EM, 9.9 Van
**25**	Vanilla Tahity	0.19	42.8	9.0 Anis, 2.8 Tri, 31 Van
**22**	Cinnamon Ceylon	0.07	26.8	26 CAD

Supplementary Table 2. Relationship between refill fluid IC_{50} data from Bahl et al. 2012, total flavor chemical concentration identified in this study, and the presence of one or more dominant flavor chemicals. Inv. # = inventory number. Total chemical flavor conc. = the average total concentration of replicate samples. TN = Tennessee. EM = ethyl maltol. Van = vanillin. CAD = cinnamaldehyde. Eug = eugenol. Tri = Triacetin. Anis = p-anisaldehyde. BPGA* = Benzaldehyde propylene glycol acetal. ***** not tested for cytotoxicity.

Chemical Name	Which cell type is more sensitive at 3V?	Which cell type is more sensitive at 5V?	Evidence of RXP at 5V? If yes, which cell type?
**Menthol**	hPF	hPF	Yes, Both
**Cinnamaldehyde**	hPF	hPF	No
**Ethyl Maltol**	hPF	hPF	No
**Eugenol**	hPF	Similar	Yes, Both
**Maltol**	hPF	Similar	No
**Vanillin**	hPF	A549	Yes, A549
**Ethyl Cinnamate**	hPF	A549	Yes, A549
**Propylene Glycol**	Similar	hPF	Yes, hPF
**Benzyl Alcohol**	Similar	Similar	No
**Benzaldehyde**	Similar	Similar	Yes, Both
***p*-Anisaldehyde**	Similar	A549	Yes, A549
**Triacetin**	Similar	A549	Yes, A549
**Menthone**	Similar	A549	No

Supplementary Table 3. Cell type sensitivities and evidence of reaction products from heating dominant flavor chemicals and propylene glycol. For cell type sensitivity, a response of 'Similar' indicates both hPF and A549 having a similar dose-response curves for a chemical. For reaction products (= RXP) evidence, a response of 'Both' indicates that hPF and A549 each had a dose-response curve shift to the left at 5V as compared to 3V for a chemical. Rows are ordered by cell type sensitivities with hPF being most sensitive, then "Similar" which includes both cell types, and lastly A549.
